# Biological resilience as a crucial determinant in preventing age-associated chronic diseases

**DOI:** 10.3389/fphys.2025.1741426

**Published:** 2025-12-17

**Authors:** Amjad Islam Amjad, Bisma Sajjad Sheikh, Sarfraz Aslam

**Affiliations:** 1 Department of Education, The University of Lahore, Lahore, Pakistan; 2 Institute of Education and Research, University of the Punjab, Lahore, Pakistan; 3 Faculty of Education and Humanities, UNITAR International University, Petaling Jaya, Malaysia

**Keywords:** ageing, autophagy, biological resilience, chronic diseases, immune system

## 1 Introduction

The world is ageing at an unprecedented rate, and by 2050, the global population is projected to have more than two billion people aged 60 years and above (Paul and Nag, 2024). This demographic transition poses a significant challenge to the healthcare systems worldwide. It will impact the healthcare system because the number of age-related chronic diseases like cardiovascular disease, diabetes, neurodegenerative conditions, and cancer grows significantly as age advances (Kopp, 2024). Earlier studies on ageing have primarily focused on the accumulation of molecular and cellular damage as the basis of these diseases.

However, the damage-centric perspective has shortcomings when explaining the heterogeneity of older adults, where not all of them are chronologically old, but are still very functional and independent. Biological resilience, that is, the body’s capacity to recover physiologically, environmentally, and psychologically when stressed by certain factors, has become a crucial predictor of healthy ageing (Guo et al., 2022). Ageing is associated with several health-related problems, as individuals are more susceptible to illnesses at this stage of life (Le Couteur and Thillainadesan, 2022). Older age is among the most common risk factors for various chronic illnesses, which is traditionally explained by the fact that there is a cumulative amount of molecular and cellular damage that occurs during the ageing process. Recent developments in ageing biology have focused on enhancing biological resilience. It is the ability to rejuvenate in the face of physiological difficulties and reestablish homeostasis (Promislow et al., 2022). It was found to be one of the most important conditions for healthy ageing and disease prevention. This opinion paper examines the role of biological resilience in managing, preventing, and slowing the progression of age-related diseases, drawing on both theoretical and empirical foundations. There is a need to understand what can be done to minimise the risks associated with ageing. This paper has therefore critiqued measurement tools, discussed how resilience affects vulnerability to disease, and examined interventions to improve resilience, health span, and longevity among the elderly.

## 2 Theoretical foundation of biological resilience

This opinion paper explores the concept of psychological resilience to establish the foundation for biological resilience and its relationship to chronic diseases. Biological resilience includes a few systems, including cell repair processes, immune responses, metabolic stability, and neuroendocrine regulation. They all operate potentially to sustain physiological balance in the presence of stressors (Jasbi et al., 2025). In addition to the damage accumulation model, resilience frameworks prioritise adaptive recovery processes. Therefore, it has also been discovered that decreases in these adaptive abilities have become a fundamental attribute of ageing and disease vulnerability (Lissek, 2024).

A multifaceted network of cellular and molecular responses forms the basis of biological resilience, which helps sustain the organism’s homeostasis (Weavers, 2024). The basis of these is the ability to repair DNA, which will alleviate the harmful effects of genomic damage accrued during metabolic activities and environmental challenges. Base and nucleotide excision repair is one of the DNA repair pathways; as the efficiency of repair processes decreases with age, it impairs cellular stability and repair capacity (Rathinam et al., 2025). The other important process is autophagy, a cellular process that recycles damaged organelles and proteins, and is necessary to maintain proteostasis, which is critical to cell function. Autophagy deterioration with age promotes the development of pathologic proteins typical of most neurodegenerative conditions (Palmer et al., 2025).

Mitochondrial dynamics, encompassing biogenesis, fusion, and fission, are crucial for both energy production and signalling (Chen et al., 2023). The age-related dysregulation of mitochondrial activity leads to a reduction in energy availability, an increase in oxidative stress, and a decline in cellular and systemic resilience. The most important aspect is also immune modulation; an adequately regulated immune system may assist the body in fighting the infection and restoring the tissues. Immunosenescence and the continued presence of low-grade inflammation (inflammaging) undermine the immune response and intensify the pathology (Fortingo et al., 2022). The regulating factors of gene expression that influence resilience capacity in response to stress and ageing are also undergoing epigenetic modifications, including DNA methylation and histone modifications (Wang et al., 2022). Most stressors activate positive adaptive pathways when subjected to low-dose stress, which is further reinforced by hormetic stress responses that stimulate cellular repair and survival signalling (Calabrese et al., 2021). Collectively, these mechanisms demonstrate how biological resilience operates as a dynamic, integrative process that is essential for maintaining health during ageing.

Resilience occurs in the physical, cellular, and psychological levels, and there are different ways to be thrown into the downward spiral. The anticipated domain-specific losses typically appear in early ageing; advanced ageing usually leads to global resilience losses (Pavlova et al., 2023). Psychological resilience influences the biopsychosocial model, as ageing is a complex process. Psychosocial resilience is a key modulator of physiological ageing patterns, in which biological and environmental factors interact extensively. The opinion paper will address the following research question, based on the above narrative.

How does biological resilience influence the prevention of age-associated chronic diseases, and what interventions can effectively preserve or enhance resilience to promote healthy ageing?

## 3 Literature review and rationale

Recent literature reviews and meta-analyses have converged on the premise that biological resilience is an exciting but poorly measured construct in ageing studies. Several studies focus on individual dimensions, including psychological resilience and individual biomarkers, but few take comprehensive frameworks that incorporate physiological, cellular, and psychological arenas. The researchers found that resilience measurement is an important topic for elderly people. It can be measured with the help of several indicators such as environment, climate, biological and psychological factors. Moreover, the psychosocial environment, including stress exposure, socioeconomic status, and lifestyle, interacts with the complex biological resilience, making it challenging to separate causal pathways. In light of these challenges, this study adopts a narrative review approach to integrate various strands of research into a cohesive framework. The objective of this opinion is to propose innovative strategies for improving biological resilience among elderly people.

## 4 Methods

The researchers employed a qualitative narrative review approach in this study. The authors searched data using various databases like PubMed and Scopus, with keywords such as “biological resilience ageing,” “resilience interventions,” and “age-associated disease prevention.” The search results have been limited to publications dated between 2020 and 2025. Empirical studies, reviews, and clinical trials of biological and psychological resiliency in ageing situations were to be incorporated to develop the inclusion criteria. Based on the chosen studies, the critical appraisal was given to the studies based on their relevance and the rigor of their methods. The thematic data synthesis was used to analyse the fundamental concepts, measurement methodology, disease associations, and intervention plans. This research design allowed us to integrate multidisciplinary results and conceptual development comprehensively and identify gaps in knowledge. Weaknesses include an examination of published secondary data without empirical data gathering.

## 5 Assessment of biological resilience

The researchers employed integrative measurements that combined biological and psychological data, thereby validating resilience profiling and intervention in older adults. As seen in the literature, the calculation of resilience is still complex due to its multidimensionality. However, the following approaches are present in the current opinion paper:

Biomarkers perceive and measure inflammation, mitochondrial activity, metabolic phenotypes, and epigenetic signals to measure physiological resilience (Furrer and Handschin, 2025). It is found that biomarker snapshots and static systems of dysregulation. Thus, it is difficult to understand how long old people will take to recover. It depends on nature of illness and stressors and cut-off points cannot be true for all people (Buller-Peralta et al., 2025; Polick et al., 2024). In this regard measures like, metabolic, allostatic load integrate cardiovascular, neuroendocrine and inflammatory can help to understand morbidity and mortality in later life (Arkesteijn et al., 2025). But there is chance of error in allostatic load indices which can limit the comparison of clinical use and previous studies (Sahab et al., 2025).

The capacity of homeostatic recovery is measured by functional testing (Corrao et al., 2024). These indicators like, gait speed, chair stands, and mental stress tests are closer to older people’s recovery and performance. These are sensitive to intrinsic capacity changes and perform better under real-life stressors (Grigoraş et al., 2025; Mailliez et al., 2025). However, these indicators can be fluctuated with short term factors like acute symptoms.

Psychological resilience measures like coping and emotional regulation functions, meaning to life, and perceived control are important (Troy et al., 2023). These indicators are of significant importance for adults. The research indicates that people who have higher psychological resilience, they survive longer even with physical stressors (Cosarderelioglu et al., 2025; Stenroth et al., 2023). However, cultural norms, current mood, and response style can affect self-reporting of biological resilience. These shortcomings provide the foundation for the integration of functional and psychological domians.

## 6 Biological resilience and preventing chronic disease

The literature suggests that a decrease in resilience mechanistically increases susceptibility to cardiovascular disease, neurodegeneration, diabetes, and frailty (Ruggiero, 2024). However, physiological insults typically recover rapidly, which restrains the development of chronic diseases. To illustrate the protective effect, psychological resilience is associated with lower total and cardiovascular mortality, which is independent (Ghulam et al., 2022). The lack of resilience is linked with a decrease in the recovery period and a negative outcome following an insult, which is why it has a prognostic role. In this way, preserving resilience does not focus on treating disease, but on maintaining physiological reserves in older adults through preventive measures. Table 1 provides information about interventions to improve biological resilience among older adults.

**TABLE 1:** Biological resilience and interventions.

Biological component	Age-associated changes	Related chronic diseases	Interventions
Stem cell reserves	Exhaustion, reduced regeneration	Frailty, impaired wound healing	Stem cell therapy, exercises
Mitochondrial function	Energy production decline	Neurodegeneration, fatigue	Mitochondrial transplantation, metformin
Immune system	Dysregulation, chronic inflammation	Cardiovascular disease, infections	Caloric restriction, senolytics
Metabolic regulation	Insulin resistance, altered nutrient sensing	Diabetes	Exercise, caloric restriction
Epigenetic stability	DNA methylation changes, genomic instability	Cancer, multiple diseases	Pharmacological epigenetic modulators

Table 1 indicates that combined caloric restriction and exercise are multi-modal interventions, which synergise to enhance metabolic, mitochondrial, and immune resilience in older adults (Chen et al., 2023). Moreover, pharmacological agents like senolytics and rapamycin analogs modulate aging pathways, enhancing resilience (Moskalev et al., 2022). Supporting pharmacological agents, emerging regenerative therapies (stem cells, mitochondrial transplantation) show significant improvement in replenishing depleted reserves (Kaye et al., 2022). Psychological resilience training is a technique that complements biological ones by regulating neuroendocrine and immune functionality (Bjekić and Petrović, 2024).

## 7 Discussion and future directions

The intervention-based evidence and literature review suggest that biological resilience is mainly focused and is determinant of vulnerability to age-related chronic diseases. These findings responded to the research question. The conceptualisation of ageing as a slow erosion of ageing resilience, supported by the analysed evidence, suggests that a decrease in adaptive capacity predisposes individuals to increased morbidity and mortality. The decrease in resilience is heterogeneous across physiological domains; some systems deteriorate sooner and faster than others, resulting in heterogeneous ageing phenotypes. This domain-specific/global resilience impairment contrast is essential in the design of interventions directed, which has not been extensively developed in previous frameworks (Ruggiero, 2024). Theoretical extensions based on this review suggest the re-conceptualisation of ageing biology as a complex and fragile network of interdependent resilience thresholds. In this networked resilience model, failures in any aspect can seep into others. The model integrates molecular damage, regulatory dysfunctions, and reserve depletion. This can be shaped with help of both internal (genetics, organ reserves, and cellular repair) and external factors (lifestyle, environmental exposures, and psychological stressors).

This networked resilience model has three core layers. For instance, at the micro level molecular and cellular processes, including DNA repair, proteostasis, mitochondrial dynamics, and epigenetic regulation set the baseline capacity. At the second level of meso, system functions and organs, including immune, metabolic, and cardiovascular, enhance the capacity into functional reserves. However, at the macro level, social support, psychological resilience, and environmental context play a decisive role in the impact of organisms (Grigoraş et al., 2025; Promislow et al., 2022). It indicates that even small losses in one layer can trigger nonlinear transitions towards frailty when the network loses adaptive flexibility.

### 7.1 Climate-related stressors and biological resilience

Climate change is a major threat to older adults. For example, air pollution, temperature variability, and extreme cold or heat influence cardiovascular and respiratory conditions (Liu et al., 2022; Siddiqui et al., 2025). Physiologically, aging reduces sweat production, vascular elasticity, cardiac reserve, and renal concentrating ability. This further impairs thermoregulation and dehydration (Núñez-Rodríguez et al., 2025). These climate-related stressors are real threat to resilience in the aged population. These stressors interact with the inflammatory and immune systems. Air pollution and heat waves can disturb autonomic regulation and low-grade inflammation. This influences neurodegeneration, frailty, and cardiovascular disease in old age (Cosarderelioglu et al., 2025). This shows that climate-related factors can significantly influence biological resilience.

However, on the other hand, climate strategies can be used to improve aging resilience as well. For example, a heat-health action plan linked with warning systems, hydration campaigns, and cooling centers reduces mortality during heat waves (Lv et al., 2025; Zhang, 2025). Similarly, in urban localities interventions such as housing insulation and green spaces can improve air quality (Grigoraş et al., 2025; Zhang, 2025). These adaptations can shift resilience threats to protecting aging physiology. The studies on environmental psychology and climate gerontology had also added the psychosocial aspect. It is also evident from the literature that aged people who engage community-based climate initiatives indicate higher psychological resilience and social connectedness (Marinova et al., 2025; Zhang and Chen, 2024). Thus, it can be said that climate-related exposures and climate action can lead to external threats and also provide opportunities to improve psychological and biological resilience in later life.

## 8 Conclusion

The researchers consider biological resilience to be a key factor to understand and help to reduce age-related chronic diseases. The current evidence indicates that resilience-related factors are of significant importance to understand and manage age-related diseases. Even though they work in the presence of several other social and biological determinants. This review shows that preserving and enhancing resilience, spanning molecular repair, systemic regulation, and psychological adaptation, is integral to maintaining health in later life. It is helpful to understand that aging is a decay process.

It was found that there should be a balance among psychological, climate-related, biological and day-to-day routine factors. The imbalance in one aspect can trigger the disturbance in others. Integrating advanced measurement techniques and innovative multi-modal interventions makes anticipating and mitigating declines before irreversible pathology ensues feasible.

We are of the opinion that the proactive strategies can be very helpful to reduce healthcare burden in aging populations. It will decrease disability rates and enhance functional independence among older individuals. If future researchers are suggested to design gerontology studies for resilience by including various factors discussed in this opinion paper using longitudinal approaches, this will help us to examine the longer-term effects of such variables in the aging community. It will also help to improve their mental, physical, and overall wellbeing. Ethical considerations, including equitable access to therapies and the societal value placed on ageing individuals, require immediate attention. Furthermore, advancing resilience science invites a future where aging is considered as sustained vitality, adaptability, and quality of life. We are of opinion that interdisciplinary research and innovative clinical interventions are essential to understand biological resilience.
